# A machine learning-based prediction model for poor prognosis in sepsis using lymphocyte count: a national, multicenter prospective cohort

**DOI:** 10.1038/s41598-025-33980-x

**Published:** 2026-01-22

**Authors:** Siang Huang, Luyao Liu, Chaoyang Wang, Xu Li, Yina Liu, Xiaochun Ma, Yini Sun

**Affiliations:** 1 Department of Critical Care Medicine, The First Hospital of China Medical University, China Medical University, 155 Nanjing North Street, Heping District, Shenyang City, 110001 Liaoning Province China; 2 State Key Laboratory for Diagnosis and Treatment of Infectious Diseases, The First Hospital of China Medical University, Shenyang, China

**Keywords:** Sepsis, Lymphopenia, Trajectory analysis, Prognosis, Biomarkers, Computational biology and bioinformatics, Diseases, Immunology, Medical research

## Abstract

Sepsis-induced immunosuppression leads to poor prognosis. Circulating lymphocyte count (LC), as an easily accessible clinical marker, closely reflects the immune status of sepsis. The study aims to perform immune phenotyping of sepsis patients using dynamic LC for early identification of high-risk individuals. A latent class trajectory model (LCTM) was used to analyze the dynamic trajectories of lymphocyte count (LC) based on repeated measurements obtained within at least two measurements of lymphocyte count (LC) within the first 24 h after sepsis diagnosis, followed by two more between day 2 and day 7. Survival differences among subphenotypes were assessed using Kaplan–Meier curves and Cox regression. Feature selection was conducted via the Boruta algorithm, and a high-precision machine learning model was developed to predict the target trajectory. Model interpretability was ensured through SHapley Additive exPlanations (SHAP). The predictive performance of the model for ICU mortality was assessed using the receiver operating characteristic (ROC) curve. The derivation cohort included 2085 sepsis patients from the China Multicenter Sepsis database, and the external validation cohort of 1299 sepsis patients. We identified four trajectory patterns of LC dynamics, among which the persistent lymphopenia (PL) subgroup exhibited the highest disease severity and poorest prognosis. The trajectory model demonstrated consistent patterns in external validation. Six machine learning models were utilized to determine the best model to identify the PL subphenotype, and an online prediction tool was developed for clinical application. Incorporating the PL trajectory subphenotype significantly improved the predictive performance for ICU mortality. Dynamic LC trajectories effectively capture immunological heterogeneity in sepsis, encompassing immunocompromised and immunocompetent hosts. These findings underscore the importance of early identification of patients with persistent lymphopenia to better target populations for future sepsis immunotherapy.

**Supplementary Information:**

The online version contains supplementary material available at 10.1038/s41598-025-33980-x.

## Introduction

Sepsis is a dysregulated host response to infection, leading to organ dysfunction and high mortality^1^. Increasing evidence suggests that pro-inflammatory activation and immunosuppression coexist from the early stages of sepsis^2^. Sepsis-induced immunosuppression is characterized by extensive lymphocyte apoptosis, downregulation of HLA-DR expression, and impaired immune function^3^. Although previous studies have attempted to treat sepsis with immunomodulatory agents, most large randomized clinical trials have failed to demonstrate significant benefit^4^. Septic patients exhibit substantial heterogeneity in the immune responses, driven by the differences in disease severity and pre-existing immune status^5,6^.

A key manifestation of sepsis-induced immunosuppression is lymphocyte apoptosis, resulting in decreased peripheral lymphocyte counts, which are consistently associated with secondary infections, persistent organ dysfunction, and increased mortality. Persistent lymphopenia has therefore emerged as a clinically relevant marker of impaired immune status^7–9^. Although previous studies have attempted to establish dynamic lymphocyte count trajectories in sepsis, most have significant limitations. For example, a prospective multicenter study excluded immunocompromised hosts and assessed lymphocyte dynamics only within the first 72 h after sepsis diagnosis^10^. Another study using the MIMIC database excluded patients with immune-related comorbidities, effectively removing approximately 50% of septic patients^11^. However, in clinical practice, many septic patients have underlying conditions such as malignancy or organ transplantation, which lead to compromised baseline immune function^12^. While persistent lymphopenia has been associated with adverse outcomes in critically ill patients^13^, our study builds upon this evidence by focusing on patients with sepsis and validating the association in a nationwide multicenter cohort.

We utilised the latent class trajectory model (LCTM) to identify distinct 7-day lymphocyte trajectories using data from a nationwide, prospective multicenter sepsis cohort. Model stability was validated in an independent external cohort. We further compared the subphenotypes regarding inflammatory responses, coagulation function, and clinical outcomes. Finally, we developed machine learning models for the early identification of the persistent lymphopenia (PL) subphenotype and created a web-based application to facilitate clinical translation. Our goal is to provide a foundation for the early recognition of patients with persistent lymphopenia, to enable precision interventions and for enhanced clinical outcomes.

## Methods

### Study design and participants

**Fig. 1:**
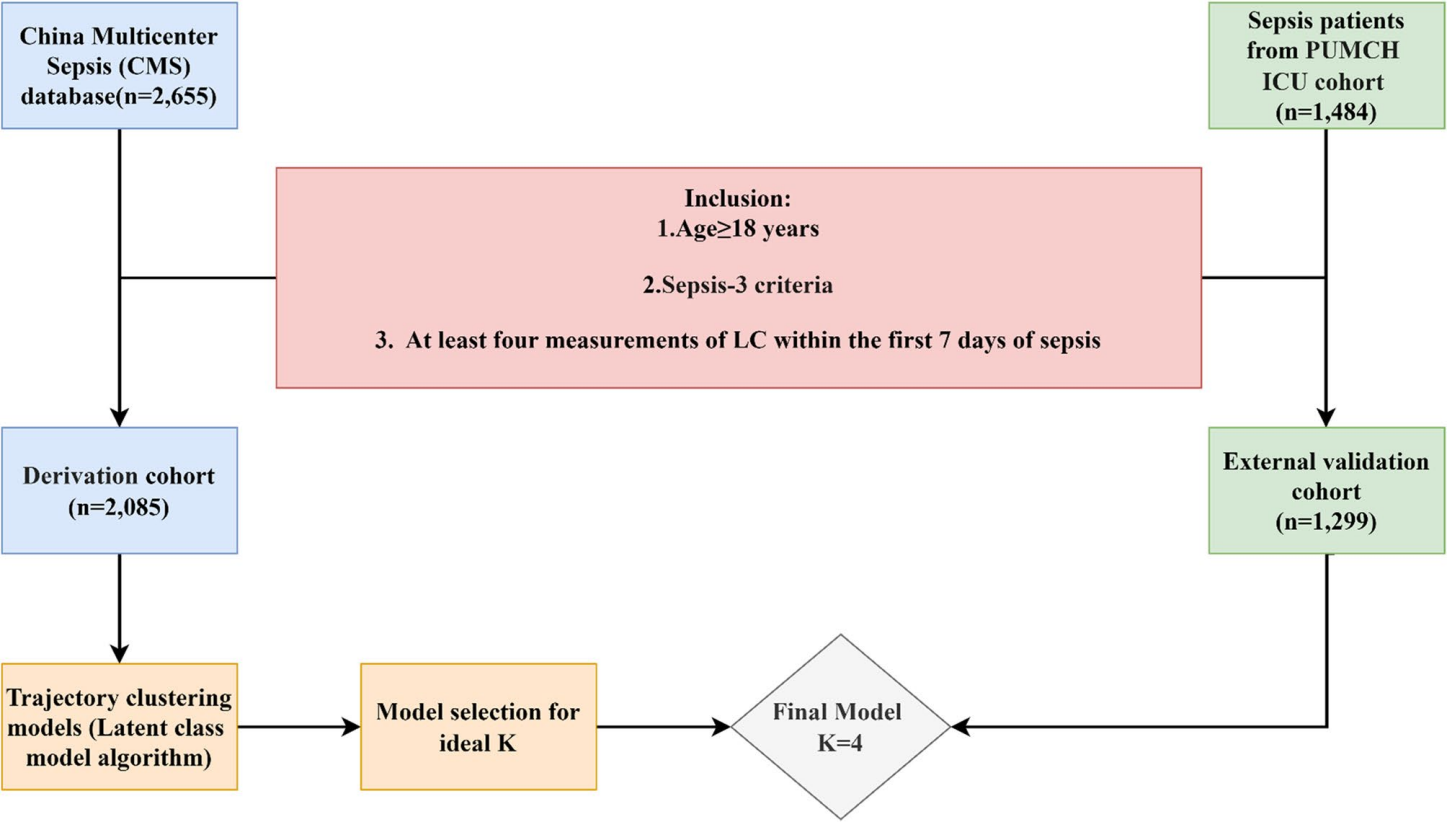
Flow diagram of participant enrollment with a dynamic modeling framework for lymphocyte count (LC) trajectories.

This study included a derivation cohort and an external validation cohort (Fig. 1). The derivation cohort was derived from the China Multicenter Sepsis (CMS) database, hosted by the First Hospital of China Medical University. The CMS database included adult septic patients from January 1, 2023, to December 31, 2024 (n = 2,655), involving 27 intensive care units (ICUs) from tertiary university hospitals (details in Supplementary Table 1). Moreover, the external validation cohort included adult sepsis patients (n = 1,484) admitted to the ICU of Peking Union Medical College Hospital from January 1, 2023, to December 31, 2024. Sepsis was defined according to the Sepsis-3 criteria^1^. The inclusion criteria were as follows: (1) aged ≥ 18 years; (2) had at least two measurements of lymphocyte count (LC) within the first 24 h after sepsis diagnosis, followed by two more between day 2 and day 7. The study has been performed in accordance with the Declaration of Helsinki and was approved by the Research and Ethics Committee of the First Affiliated Hospital of China Medical University (Approval number: [2022] 2022–502-2, Shenyang, China) and the Institutional Review Board of Peking Union Medical College Hospital (Approval number: JS-3480D). The other 26 participating ICUs obtained their respective ethical approvals. Written informed consent was obtained from all participants and/or their legal surrogates before enrollment.

### Data collection

Baseline data included demographic characteristics (age, sex), site of infection (lung, abdomen, urinary, bloodstream, skin, nervous system, and others), and preexisting conditions (hypertension, diabetes, coronary heart disease, chronic obstructive pulmonary disease, cancer, and immunocompromised). Immunocompromised hosts were defined by the presence of any of the following conditions: (a) acquired immunodeficiency syndrome; (b) malignant neoplasms treated with radiotherapy or chemotherapy within the preceding three months; (c) receipt of allogeneic bone marrow or hematopoietic stem cell transplantation; (d) receipt of solid organ transplantation; (e) autoimmune disease or ongoing immunosuppressive therapy; (f) glucocorticoid therapy (prednisolone > 20 mg/day or equivalent for > 2 weeks) within the preceding three months; and (g) chronic viral hepatitis. In the derivation and external validation cohort analyses, the initial measurements of vital signs and laboratory tests within the first 24 h of sepsis diagnosis were recorded. The laboratory variables included white blood cell count (WBC), LC, procalcitonin (PCT), C-reactive protein (CRP), platelet count (PLT), prothrombin time (PT), international normalized ratio (INR), activated partial thromboplastin time (APTT), fibrinogen (Fg), D-dimer (DD), fibrin degradation products (FDP), the PaO_2_/FiO_2_ ratio, lactic acid (Lac), creatinine (Cr), and total bilirubin (TBIL). The Acute Physiology and Chronic Health Evaluation (APACHE) II score, Sequential Organ Failure Assessment (SOFA) score, and outcomes were recorded.

### Latent class trajectory model

The Latent Class Trajectory Model (LCTM)^14^ is a finite mixture model for longitudinal data. It groups individuals into hidden (“latent”) classes based on similar change patterns over time and fits a polynomial regression within each class to model their trajectory. The optimal number of classes was determined using a lower Akaike information criterion (AIC), Bayesian information criterion (BIC), and higher entropy, indicating a better model fit (Supplementary Table 4). Additionally, to increase the clinical relevance and reliability of the statistical analysis, a minimum subgroup size of 2% of the total cohort was established, along with the average posterior probability of group membership ≥ 70% for all subphenotypes^15,16^. Furthermore, we balanced model performance with clinical interpretability to select the final trajectory model. The chosen model was subsequently evaluated on the external validation cohort. Individual LC data from each patient in the validation cohort were fitted to the polynomial mixture components of the trained LCTM.

### Model construction for the identification of subphenotype 1

We first selected clinical parameters that showed significant differences in subphenotype comparisons. The Boruta algorithm^17^ was then employed to determine the final feature set for modeling by comparing their importance to permuted “shadow” features, iteratively confirming or rejecting features for optimization. The derivation cohort was randomly split into training and test sets at a 7:3 ratio. Subsequently, six machine learning models, including logistic regression (LR), random forest (RF), support vector machine (SVM), extreme gradient boosting (XGB), multilayer perceptron (MLP), and light gradient boosting machine (LightGBM), were used to predict subphenotype 1 in sepsis patients. For each model, hyperparameter tuning was conducted using 10 rounds of tenfold cross-validation combined with Bayesian optimization based on the selected feature subset. The area under the receiver operating characteristic curve (AUC) was calculated for each model performance evaluation. Additionally, calibration curves and decision curve analysis (DCA) were used to evaluate the model’s performance. The Shapley Additive Explanations (SHAP) algorithm was also used for model interpretation. It ranks how vital each input feature is and clarifies predictions using SHAP values. To facilitate clinical translation, we developed a web-based calculator using a Streamlit application to identify Subphenotype 1 based on the best model.

### Statistical analysis

Outliers in continuous variables were first screened using box plot analysis. An observation was considered an outlier if it exceeded the upper quartile (Q3) by more than 1.5 times the interquartile range (IQR) or fell below the lower quartile (Q1) by the same criterion. Identified outliers were then addressed using winsorization, in which extreme values were replaced with the corresponding upper or lower boundary defined by the 1.5 × IQR rule^18^. This procedure minimized the influence of extreme values while preserving the overall sample size. For the LC trajectories, no missing data processing was required because the LCTM inherently accommodates missing values through maximum likelihood estimation during model fitting. For the clinical characteristics, the disappearance rate for variables was calculated and reported (Supplementary Table 2). To ensure the accuracy of the results, we excluded variables with missing rates over 30%^19^. Based on this criterion, only Fibrin Degradation Products (FDP) were removed.

Continuous variables were presented as median (interquartile range [IQR]) and compared across subphenotypes using one-way ANOVA (if assumptions of normality and homogeneity of variance were met) or the Kruskal–Wallis test otherwise. Categorical variables, expressed as counts (percentages), were compared using the chi-squared test. We compared 28-day mortality in the derivation and validation cohorts via Kaplan‒Meier plots and estimated hazard ratios (HRs) across subphenotypes using Cox proportional-hazards regression models.

Independent predictors of ICU mortality were identified through univariable and multivariable logistic regression analyses. Two prediction models were constructed: a baseline model containing only conventional risk factors and an enhanced model incorporating the trajectory of persistent lymphopenia (PL). The AUCs of the two models were compared using DeLong’s test, with *p* < 0.05 considered statistically significant. All analyses were conducted in R (v4.3.0) and Python (v3.12.7). Key packages included: R: ‘lcmm’(v2.2.0) for latent class trajectory modeling, ‘mice’ (v3.16.0) for multiple imputation, ‘Boruta’ (v8.0.0) for feature selection. Python: ‘scikit-learn’ (v1.5.1) for model development, ‘scikit-optimize’ (v0.10.2) for hyperparameter tuning; ‘xgboost’ (v3.0.2) and ‘lightgbm’ (v4.6.0) for gradient-boosted classifiers. ‘shap’(v0.48.0) for model explanation.

## Result

### Patient Characteristics between subphenotypes in derivation and external validation cohorts

2,085 patients from CMS were included in the derivation cohort, and 1,299 patients from PUMCH were included in the external validation cohort according to the inclusion criteria (Fig. 1). Baseline characteristics and outcomes are detailed in Supplementary Table 3.

**Fig. 2:**
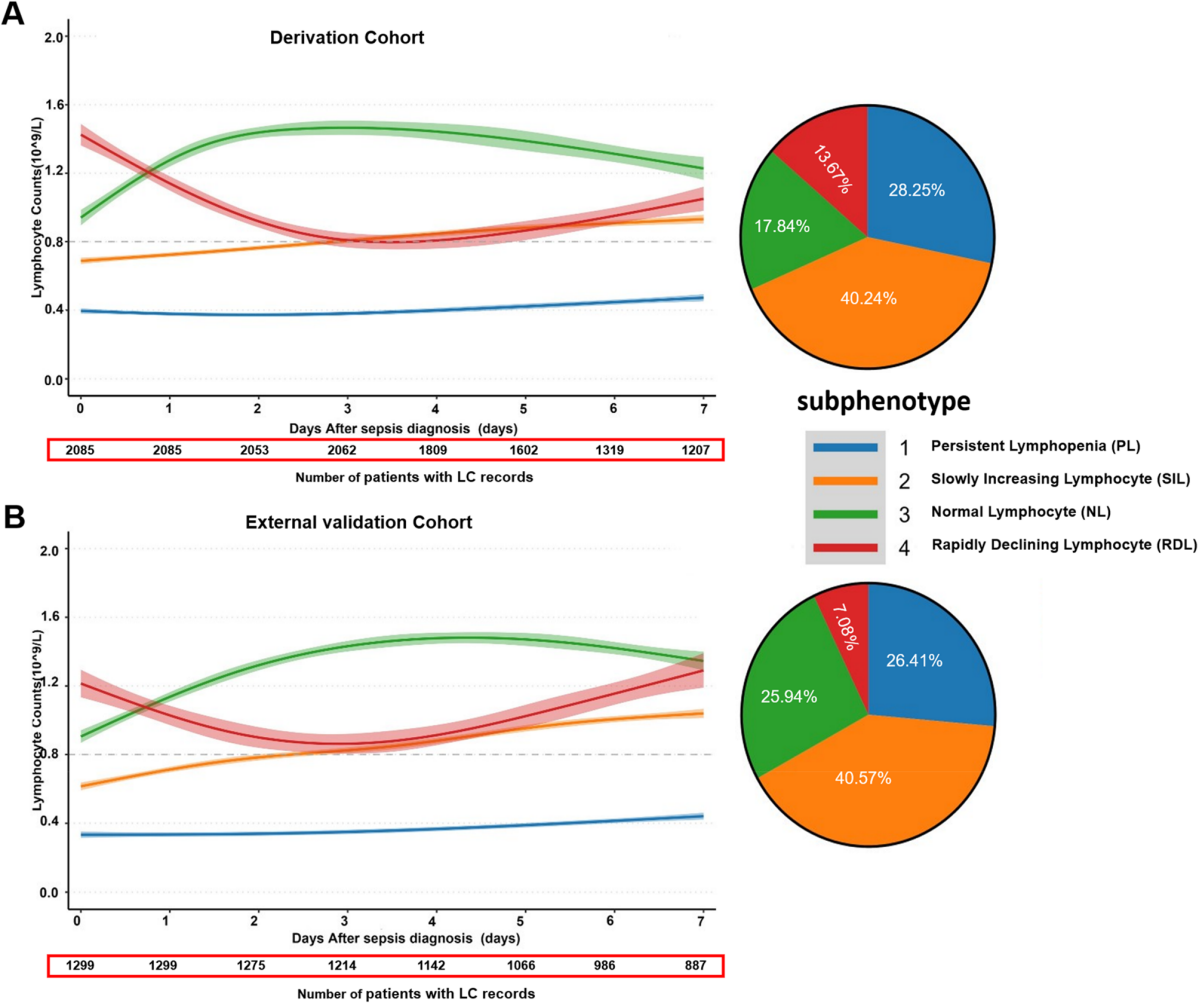
Latent Class Trajectory Model of lymphocyte count (LC) trajectories in the derivation and external validation cohorts. (**A**) The LC trajectories and the pie chart demonstrate the proportions of four subphenotypes in the derivation cohort. (**B**) The LC trajectories and the pie chart demonstrate the proportions of four subphenotypes in the external validation cohort. The shaded area of the curve represents each trajectory’s 95% confidence interval. Subphenotype 1 represents the persistent lymphopenia (PL, colored in blue), subphenotype 2 represents a low baseline LC followed by slowly increasing lymphocyte (SIL, colored in yellow), subphenotype 3 represents normal lymphocyte (NL, colored in green), and subphenotype 4 shows a normal initial lymphocyte count, followed by a rapid decline (RDL, colored in red).

Based on the model fit statistics from LCTM with 1 to 6 classes (Supplementary Table 4). The optimal four-class model with fixed effect coefficients identified four lymphocyte trajectory subphenotypes in the derivation and external validation cohorts (Fig. 2 and Supplementary Table 5):

PL (Persistent Lymphopenia, n = 589, 28.21%): Started with an initial LC count < 0.8 × 10⁹/L and remained persistently low.

SIL (Slowly Increasing Lymphocytes, n = 839, 40.26%): Started with a low baseline LC count (< 0.8 × 10^9^) followed by a slow increase to the normal range (0.8–3.2 × 10^9^).

NL (Normal Lymphocytes, n = 372, 17.65%): Maintained LC counts within the normal range (0.8–3.2 × 10⁹/L).

RDL (Rapidly Declining Lymphocytes, n = 285, 13.68%): Had an initial normal LC count with a rapid decline.

The Clinical characteristics among subphenotypes in both cohorts are presented in Table 1. Compared to other subphenotypes, PL patients were the oldest, had a higher incidence of pre-existing immunocompromised status and pulmonary infections, and a lower incidence of urinary tract infections. Correspondingly, PL had the highest SOFA and APACHE II scores, as well as the highest ICU and 28-day mortality. In contrast, NL patients were the youngest, with the mildest disease severity and the lowest mortality. Consistent with the derivation cohort, the external validation cohort showed similar demographic patterns and clinical outcomes across all subphenotypes.

**Table 1:** Comparison of clinical characteristics between subphenotypes of patients with sepsis in the derivation and external validation cohorts.

	Derivation cohort (n = 2085)					External validation cohort (n = 1299)				
Demographic characteristics	PL (n = 589)	SIL(n = 839)	NL (n = 372)	RDL (n = 285)	*p*	PL (n = 343)	SIL (n = 527)	NL (n = 337)	RDL (n = 92)	*p*
Sex (male), n (%)	396 (67.2)	571 (68.1)	211 (56.7)	173 (60.7)	< 0.001	202 (59.1)	344 (65.4)	203 (60.4)	61 (66.3)	0.187
Age (year), median (IQR)	69.0 (59.0, 76.0)	66.0 (55.2, 73.0)	64.0 (52.0, 72.0)	66.0 (56.0, 74.0)	< 0.001	65.0 (54.0, 73.0)	62.0 (50.0, 73.0)	55.0 (40.0, 68.2)	61.5 (48.0, 67.2)	< 0.001
Preexisting conditions, n (%)										
Hypertension	234 (39.7)	317 (37.8)	157 (42.2)	134 (47.0)	0.042	154 (44.9)	257 (48.8)	143 (42.4)	34 (37.0)	0.099
Diabetes	149 (25.3)	207 (24.7)	109 (29.3)	87 (30.5)	0.124	100 (29.2)	151 (28.7)	98 (29.1)	33 (35.9)	0.568
Coronary heart disease	93 (15.8)	105 (12.5)	56 (15.1)	48 (16.8)	0.186	64 (18.7)	110 (20.9)	75 (22.3)	19 (20.7)	0.711
Chronic obstructive pulmonary disease	27 (4.6)	21 (2.5)	7 (1.9)	10 (3.51)	0.064	9 (2.62)	22 (4.2)	6 (1.8)	2 (2.2)	0.237
Cancer	78 (13.2)	100 (11.9)	36 (9.7)	24 (8.4)	0.122	99 (28.9)	118 (22.4)	56 (16.6)	15 (16.3)	0.001
Immunocompromised	110 (18.7)	87 (10.4)	52 (14.0)	44 (15.4)	< 0.001	80 (23.3)	47 (8.9)	28 (8.3)	6 (6.5)	< 0.001
Infection of site, n (%)										
Lung	280 (47.5)	324 (38.6)	164 (44.1)	126 (44.2)	0.006	222 (64.7)	274 (52.0)	145 (43.0)	52 (56.5)	< 0.001
Abdomen	254 (43.1)	375 (44.7)	121 (32.5)	117 (41.1)	< 0.001	72 (21.0)	138 (26.2)	75 (22.3)	28 (30.4)	0.127
Urinary	19 (3.2)	61 (7.3)	33 (8.9)	13 (4.6)	< 0.001	8 (2.3)	18 (3.4)	26 (7.7)	2 (2.2)	0.003
Bloodstream	3 (0.5)	5 (0.6)	7 (1.9)	1 (0.4)	0.815	14 (4.1)	9 (1.7)	7 (2.1)	2 (2.2)	0.167
Skin	13 (2.2)	34 (4.0)	27 (7.2)	8 (2.8)	0.252	8 (2.3)	17 (3.2)	18 (5.3)	1 (1.0)	0.113
Nervous system	7 (1.2)	10 (1.2)	4 (1.1)	1 (0.3)	0.719	4 (1.2)	11 (2.1)	9 (2.7)	3 (3.3)	0.379
Others	15 (2.3)	30 (3.6)	16 (4.3)	19 (6.6)	0.006	15 (4.4)	60 (11.4)	57 (16.9)	4 (4.4)	< 0.001
Clinical scores, median (IQR)										
SOFA	8 (5, 10)	7 (5, 10)	7 (4, 9)	8 (5, 10)	0.002	9 (6, 12)	8 (5, 11)	7 (5, 10)	8 (6, 11)	< 0.001
APACHE II	15 (11, 18)	14 (11, 18)	13 (10, 17)	16 (12, 19)	< 0.001	22 (17, 27)	18 (13, 23)	16 (12, 21)	21 (15.8, 27.5)	< 0.001
Clinical outcomes, n (%)										
ICU mortality	181 (30.7)	151 (18.0)	63 (16.9)	65 (22.8)	< 0.001	118 (34.4)	68 (12.9)	29 (8.6)	18 (19.6)	< 0.001
Hospital mortality	197 (33.4)	167 (19.9)	71 (19.1)	77 (27.0)	< 0.001	128 (37.3)	81 (15.4)	35 (10.4)	19 (20.7)	< 0.001
28-day mortality	181 (30.7)	161 (19.2)	65 (17.5)	76 (26.7)	< 0.001	90 (26.2)	69 (13.1)	25 (7.4)	16 (17.4)	< 0.001

Presented are the comparisons of demographics, preexisting conditions, clinical scores, and outcomes between subphenotypes: PL (Persistent Lymphopenia), SIL (Slowly Increasing Lymphocyte), NL (Normal Lymphocyte), and RDL (Rapidly Declining Lymphocyte) in derivation and external validation cohorts. Age, SOFA, and APACHE II are presented as medians, and all other values are given as percentages. SOFA: Sequential Organ Failure Assessment score, APACHE II: The Acute Physiology and Chronic Health Evaluation II score. *P*-values signify the results of comparisons between subphenotypes

### Association of subphenotypes with coagulation and inflammation variables

**Fig. 3:**
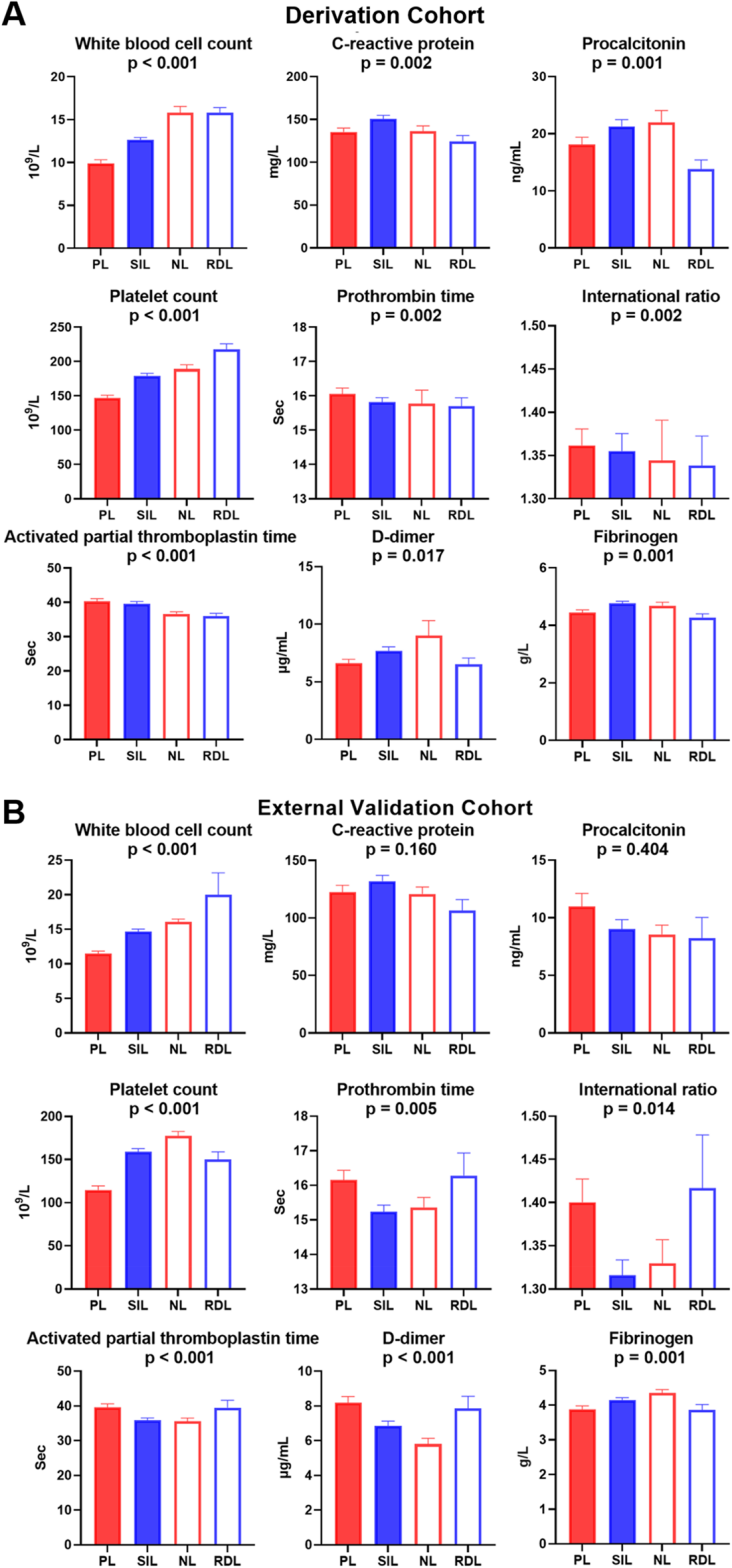
Laboratory parameters demonstrating differences across four subphenotypes are presented in the derivation cohort (**A**) and external validation cohort (**B**). The levels of laboratory parameters represent the mean and standard error of the mean (SEM). *p* values are derived from Kruskal–Wallis testing for significant differences between subphenotypes for laboratory values.

Several laboratory variables were significantly different among the four subphenotypes. As for coagulation, PL patients had the lowest PLT counts and more prolonged PT, INR, and APTT. Although PL patients exhibited the lowest WBC and LC counts, they didn’t have a higher inflammatory response. By contrast, the SIL subphenotype showed the highest inflammation indicators, PCT and CRP (Fig. 3A, Supplementary Table 6). The laboratory values in the external validation cohort generally followed a similar pattern (Fig. 3B, Supplementary Table 7).

### Clinical outcomes across subphenotypes

**Fig. 4:**
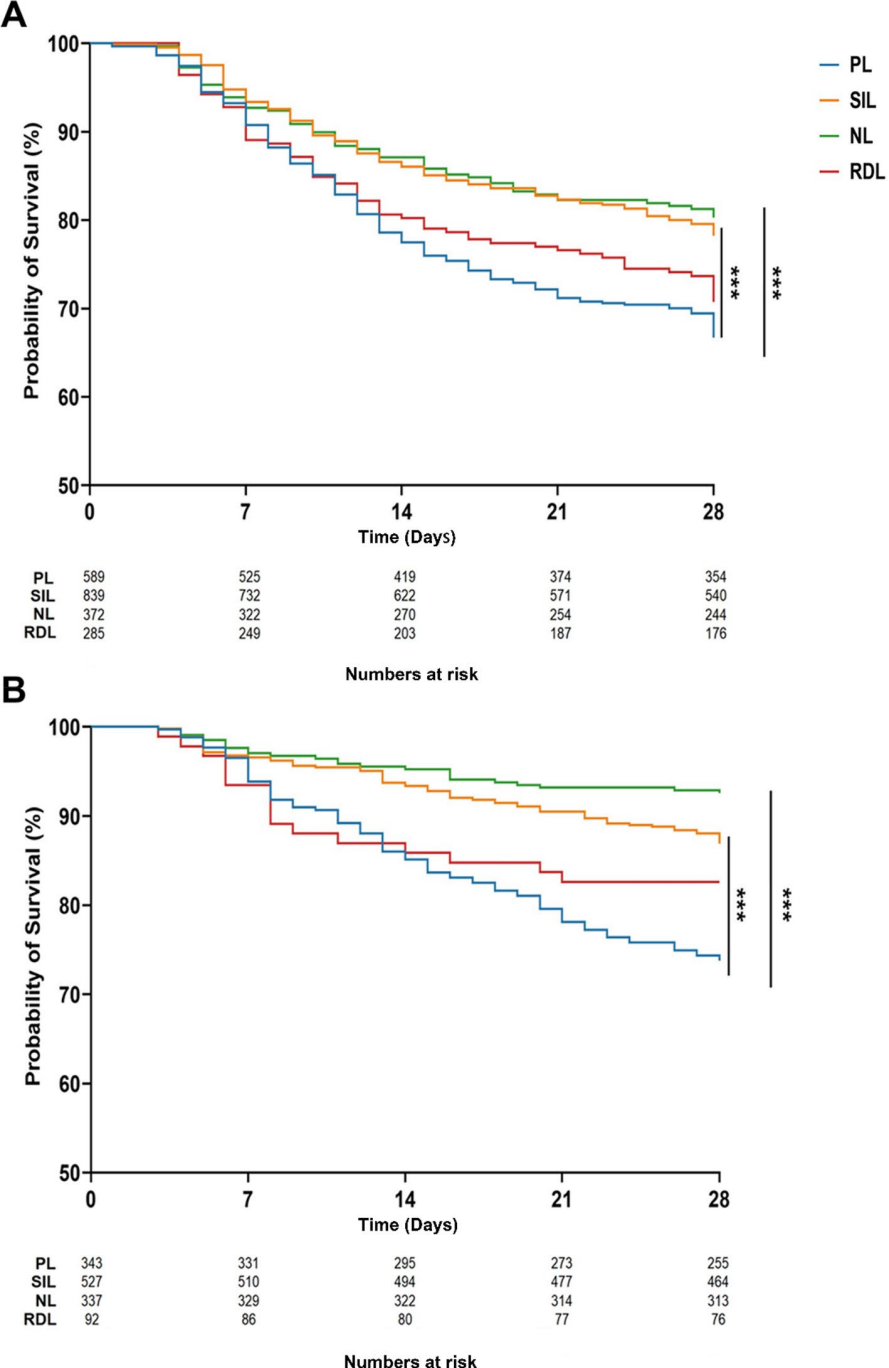
Kaplan–Meier survival curves of the four subphenotypes in the derivation cohort (**A**) and external validation cohort (**B**). PL patients have the highest 28-day mortality among the subphenotypes, while NL patients have the lowest 28-day mortality. Statistical significance was determined by the log-rank test (***p < 0.001).

To assess the association of subphenotypes with the clinical outcomes, the Kaplan–Meier curve demonstrated that PL patients had the highest 28-day mortality, while NL patients had the lowest. Compared to PL, both SIL and NL subphenotypes were associated with a significantly lower mortality, whereas RDL showed no difference in either cohort (Fig. 4). Cox regression analysis confirmed these survival differences. In unadjusted models, with PL as reference, SIL and NL were associated with significantly reduced hazard ratios for 28-day mortality, while RDL showed no significant difference. After adjusting for age, sex, SOFA, APACHE II, Immunocompromised status, and pulmonary infection, SIL and NL remained at a significantly lower risk of mortality than PL. In contrast, RDL remained statistically similar to PL. The external validation cohort confirmed these findings (Table 2A).

**Table 2:** Association of subphenotypes with ICU mortality and 28-day mortality.

28-day mortality	PL	SIL		NL		RDL	
		HR (95%CI)	*p*	HR (95%CI)	*p*	HR (95%CI)	*p*
(A)							
Derivation cohort							
Unadjusted	Reference	0.61 (0.49–0.75)	< 0.001	0.55 (0.41–0.73)	< 0.001	0.86 (0.66–1.13)	0.283
Adjusted	Reference	0.71 (0.57–0.88)	0.002	0.67 (0.50–0.89)	0.005	0.92(0.70–1.20)	0.520
External validation cohort							
Unadjusted	Reference	0.46 (0.34–0.63)	< 0.001	0.26 (0.17–0.40)	< 0.001	0.65 (0.38–1.11)	0.116
Adjusted	Reference	0.52 (0.36–0.74)	< 0.001	0.34 (0.20–0.57)	< 0.001	0.61 (0.33–1.13)	0.118

ICU mortality	PL	SIL		NL		RDL	
		HR (95%CI)	*p*	HR (95%CI)	*p*	HR (95%CI)	*p*
(B)							
Derivation cohort							
Unadjusted	Reference	0.69 (0.56–0.86)	< 0.001	0.61 (0.46–0.82)	< 0.001	0.86 (0.65–1.14)	0.313
Adjusted	Reference	0.75 (0.61–0.94)	0.012	0.76 (0.57–0.99)	0.048	0.90 (0.68–1.20)	0.491
External validation cohort							
Unadjusted	Reference	0.62 (0.47–0.89)	0.007	0.50 (0.33–0.77)	0.001	0.70 (0.41–1.19)	0.187
Adjusted	Reference	0.62 (0.44–0.87)	0.006	0.49 (0.30–0.80)	0.004	0.64 (0.35–1.16)	0.141

Multivariate Cox regression was adjusted by age, sex, SOFA, APACHEII, Immunocompromised status and pulmonary infection. Abbreviations: SOFA: Sequential Organ Failure Assessment score, APACHE II: The Acute Physiology and Chronic Health Evaluation II score, HR: hazard ratio, PL: Persistent Lymphopenia, SIL: Slowly Increasing Lymphocyte, NL: Normal Lymphocyte, RDL: Rapidly Declining Lymphocyte.

Consistent with 28-day mortality, ICU mortality was significantly lower in the SIL and NL subphenotypes than in PL, but not in RDL (Table 2B). PL patients showed minimal improvement in the SOFA score compared to the other subphenotypes (Supplementary Fig. 1).

### Identification of the subphenotype 1 (PL) among sepsis using multiple machine-learning models

**Fig. 5:**
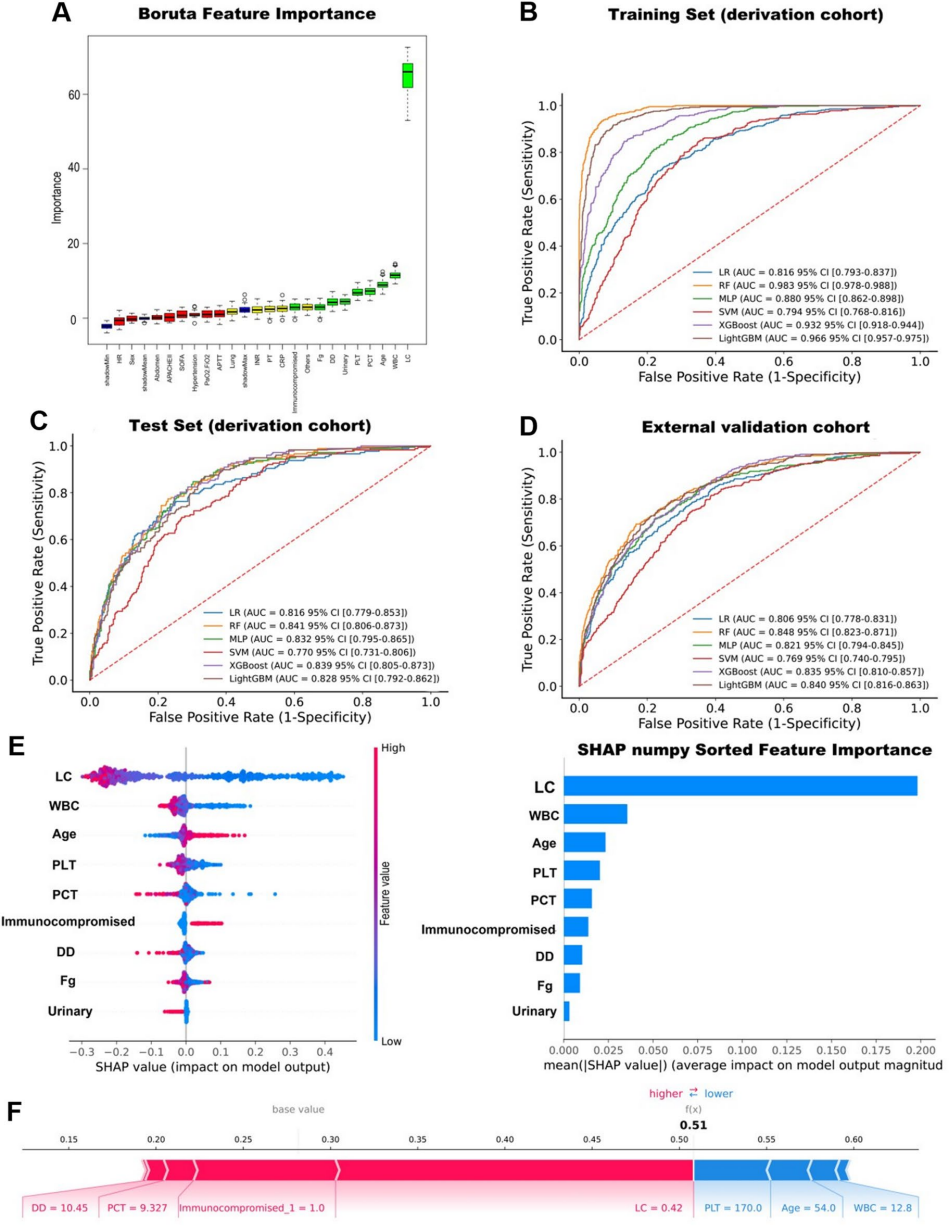
(**A**) Feature selection process for early identification of PL (subphenotype 1) patients with sepsis using Boruta’s algorithm. The x-axis displays the variables, and the y-axis shows the Z-values for each variable. Green boxplots indicate confirmed features (significantly higher importance than the maximum shadow feature), red boxplots show rejected features, and yellow boxplots represent tentative features. Blue boxplots depict the min/mean/max range of shadow features. (**B**,**C**) ROC curves compare six predictive models in the Training Set and Test Set within the derivation cohort. (**D**) ROC curves for the external validation cohort. (**E**) The characteristic attributes and importance ranking of features in the Random Forest model are shown. The x-axis shows SHAP values, and each line represents a feature. Red dots indicate higher SHAP values, while blue dots indicate lower ones. (**F**) Explanation of the prediction results for a patient with persistent lymphopenia.

Given that persistent lymphopenia (PL) septic patients exhibit the poorest clinical outcomes, we aimed to identify this high-risk subphenotype early. The Boruta strategy was applied to select features to predict PL patients in sepsis. The chosen features included LC, WBC, Age, PCT, PLT, Urinary, DD, Fg, and preexisting immunocompromised status (Fig. 5A). Six machine learning models were employed using the selected features to identify the PL patients, and the hyperparameters of each model were tuned using Bayesian optimization (Supplementary Table 8). Integrating the selected features across the training, test, and external validation sets confirmed the superior predictive performance of the Random Forest (RF) model, which was therefore selected as the final model. It achieved AUROCs of 0.983 (training set), 0.841 (test set), and 0.848 (external validation set), outperforming other models in stability and accuracy (Fig. 5B–D, Supplementary Table 9). Additionally, calibration curves and decision curve analysis (DCA) were performed (Supplementary Fig. 2A-B).

We applied SHAP to interpret the optimal prediction model and identify the most influential features. The SHAP swarm and summary bar plots indicated that lower LC, WBC, PLT, and PCT, along with older age and immunocompromised status, were the six most important features for distinguishing subphenotype 1 (Fig. 5E and Supplementary Fig. 2C). An explanation of the prediction for a specific instance of a persistent lymphopenia patient was shown in Fig. 5F. We implemented the visualization and basic application of the prediction model through a deployable web platform (https://rf-model-gf6efgvsvmcwrsj2i6cer9.streamlit.app/) using Streamlit and uploaded the source code to GitHub (Supplementary Fig. 3).

### Identification of subphenotype 1 enhanced the predictive performance for ICU mortality

**Fig. 6:**
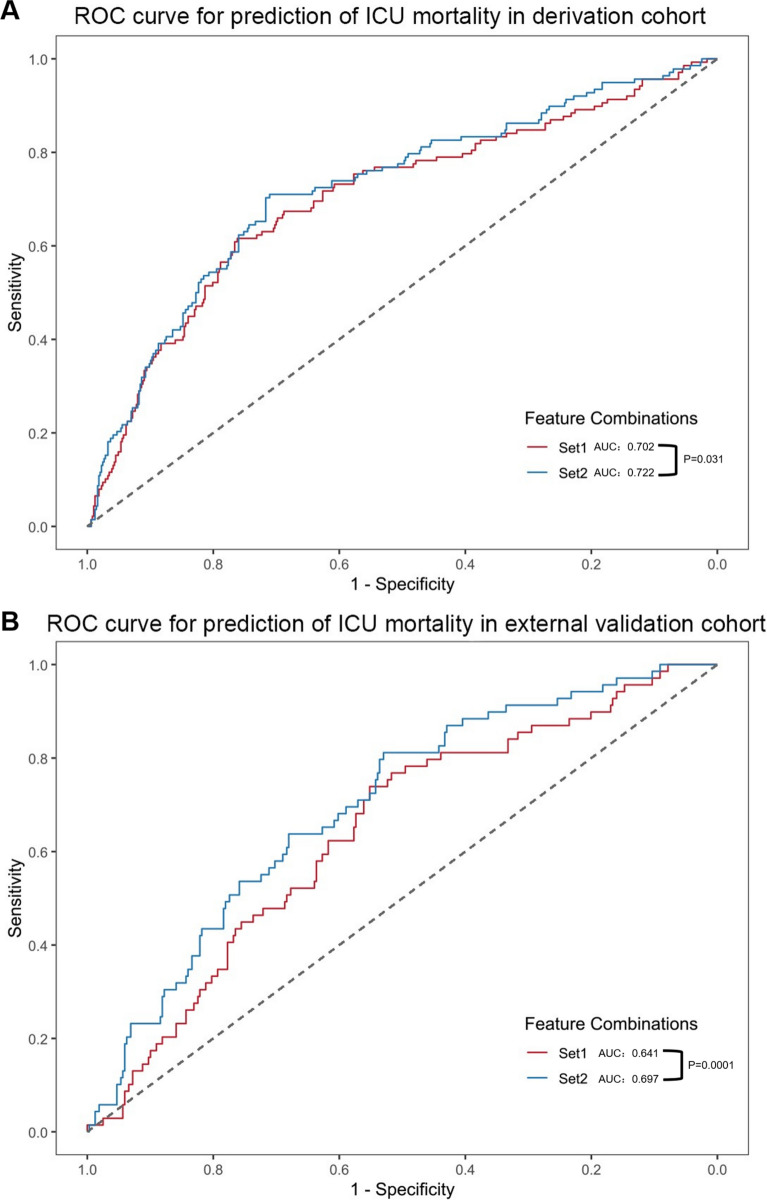
Comparison of ROC curves for ICU mortality prediction in the derivation cohort (**A**) and external validation cohort (**B**). The red curve represents the baseline model (Set1), which utilizes selected features, including pulmonary infection, Immunocompromised, Age, Temperature, Lac, and SOFA, as determined by both univariate and multivariate analyses. The blue curve illustrates the enhanced model (Set2), which incorporates the PL trajectory into the baseline model. The DeLong test compares the AUCs of the two models, revealing a significant improvement in predictive performance for the enhanced model over the baseline model in the derivation cohort (*p* = 0.031) and external validation cohort (*p* = 0.0001).

Given that PL patients had the worst outcomes, early identification of this high-risk subphenotype could be valuable for predicting clinical outcomes**.** Univariate and multivariate logistic regression analyses identified age, SOFA score, Lac, Temperature, infection source from the lung, and immunocompromised status (set1) as independent risk factors for ICU mortality (Table 3). The predictive performance for ICU mortality was significantly improved by the addition of the PL subphenotype to Set 1 (AUC increased from 0.702 to 0.722; *p* = 0.031; Fig. 6A). In the external validation cohort, the model’s AUC risen from 0.641 to 0.697 after incorporating the PL subphenotype (*p* = 0.0001; Fig. 6B). Although statistically significant, this improvement in discrimination was modest.

**Table 3:** Univariate and multivariate logistic regression analysis of the prediction of ICU mortality in the derivation cohort.

Variables	Univariate		Multivariate	
	OR (95% CI)	*p* value	OR (95%CI)	*p* value
Sex	1.06 (0.85–1.32)	0.585		
Hypertension	1.07 (0.87–1.33)	0.502		
Diabetes	1.09 (0.87–1.38)	0.457		
Coronary heart disease	1.59 (1.21–2.09)	< 0.001	1.30 (0.96–1.76)	0.090
Chronic obstructive pulmonary disease	2.13 (1.27–3.56)	0.004	1.59 (0.92–2.78)	0.099
Cancer	1.31 (0.97–1.79)	0.082		
Lung	2.31 (1.87–2.85)	< 0.001	**2.30 (1.34–3.95)**	**0.003**
Abdomen	0.60 (0.48–0.74)	< 0.001	0.99 (0.57–1.70)	0.961
Urinary	0.46 (0.27–0.80)	0.006	0.67 (0.31–1.43)	0.302
Bloodstream	0.55 (0.16–1.88)	0.345		
Skin	0.23 (0.03–1.77)	0.160		
Nervous system	0.32 (0.15–0.70)	0.004	0.61 (0.23–1.57)	0.301
Others	0.81 (0.46–1.43)	0.467		
Immunocompromised	2.14 (1.64–2.79)	< 0.001	**1.95 (1.46–2.61)**	** < 0.001**
Age	1.02 (1.01–1.02)	< 0.001	**1.01 (1.00–1.02)**	**0.002**
HR	1.00 (1.00–1.01)	0.04	1.00 (1.00–1.01)	0.245
RR	1.01 (0.99–1.02)	0.263		
MAP	0.99 (0.99–1.00)	0.017	1.00 (0.99–1.00)	0.451
T	0.86 (0.77–0.96)	0.006	**0.85 (0.75–0.96)**	**0.008**
WBC	1.00 (0.99–1.01)	0.781		
LC	0.95 (0.87–1.03)	0.201		
PCT	1.00 (0.99–1.00)	0.051		
CRP	1.00 (1.00–1.00)	0.899		
PLT	1.00 (1.00–1.00)	0.002	1.00 (1.00–1.00)	0.363
PT	1.03 (1.01–1.06)	0.006	0.98 (0.94–1.02)	0.245
INR	1.48 (1.21–1.81)	< 0.001	1.40 (0.99–1.99)	0.056
APTT	1.01 (1.00–1.02)	< 0.001	1.00 (1.00–1.01)	0.150
Fg	0.93 (0.89–0.98)	0.006	0.96 (0.92–1.02)	0.168
DD	1.00 (0.99–1.01)	0.768		
FDP	1.00 (1.00–1.00)	0.552		
PaO2/FiO2	1.00 (1.00–1.00)	< 0.001	1.00 (1.00–1.00)	0.154
Lac	1.16 (1.11–1.21)	< 0.001	**1.13 (1.07–1.19)**	** < 0.001**
Cr	1.00 (1.00–1.00)	0.207		
TBIL	1.00 (1.00–1.00)	0.053		
SOFA	1.11 (1.08–1.14)	< 0.001	**1.06 (1.02–1.11)**	**0.002**
APACHE II	1.04 (1.03–1.06)	< 0.001	1.01 (0.99–1.04)	0.222

Univariate and multivariate logistic analyses were conducted on the derivation cohort to identify predictors associated with mortality. HR: heart rate, RR: respiratory rate, MAP: mean arterial pressure, T: temperature, WBC: white blood cell count, LC: lymphocyte count, PCT: procalcitonin, CRP: C-reactive protein, PLT: platelet count, PT: prothrombin time, INR: international normalized ratio, APTT: activated partial thromboplastin time, Fg: fibrinogen, DD: d-dimer, FDP: fibrin degradation products, PaO2/FiO2: oxygenation index, Lac: lactic acid, Cr: creatinine, TBIL: total bilirubin, SOFA: Sequential Organ Failure Assessment score, APACHE II: The Acute Physiology and Chronic Health Evaluation II score, OR: odds ratio.

Significant values are in [bold].

## Discussion

This study, based on data from the China Multicenter Sepsis (CMS) database, used latent class trajectory modeling (LCTM) to describe dynamic changes in peripheral blood lymphocytes among patients with sepsis. External validation in an independent cohort from Peking Union Medical College Hospital further confirmed the reproducibility of our findings. We identified four distinct immune subphenotypes based on LC trajectories, revealing significant immunological heterogeneity in sepsis. Among them, patients with persistent lymphopenia (PL) exhibited the highest rates of immunosuppression, more frequent pulmonary infections, greater disease severity, and the poorest outcomes. We developed multiple machine learning models, among which the random forest performed best. SHAP analysis established the clinical interpretability of this model. To translate this into practice, we created an online tool to identify high-risk PL patients early. The incorporation of PL trajectory significantly enhanced the prognostic accuracy for ICU mortality.

Previous studies have assessed immune status using cross-sectional lymphocyte counts^20^, which have also observed poor outcomes in patients with sustained lymphopenia, further supporting the link between persistent lymphocyte loss and higher mortality. However, this method does not capture the dynamic change of the immune system, which can vary significantly with disease progression. In this study, we aimed to address this limitation by using a trajectory modeling approach to analyze longitudinal changes in lymphocyte counts. The patients with persistent lymphopenia indicated ongoing immunosuppression, consistent with the poor prognosis. These results align with a 2022 single-center study that identified lymphocyte trajectory subphenotypes and found the worst outcomes in the PL group^13^. However, that study included a general ICU population from a single center and did not stratify patients by reasons for ICU admission. Our study addressed these limitations by focusing on sepsis patients and validating lymphocyte count trajectories in a nationwide multicenter cohort with an independent external cohort.

Recent studies suggest that immune responses in sepsis are dynamic and potentially modifiable by anti-inflammatory treatment, showing that blood immune endotypes in COVID-19 pneumonia frequently shift over time and can be favorably influenced by targeted immunotherapy^21^. Within this framework, our lymphocyte trajectory classes likely reflect an evolving immune state shaped by both underlying host condition and intensive care interventions. Future work integrating longitudinal immunoregulatory treatment information and immune phenotyping may further clarify how specific therapies influence lymphocyte trajectories and their prognostic significance.

Notably, our findings differed from two previous multicenter studies that reported alternative prognostic patterns^10,11^. A prior single-center investigation developed lymphocyte trajectory classes and reported that patients with a “rapidly decreasing” trajectory had the worst outcomes^10^. However, their trajectories were derived from a single-center cohort, and immunosuppressed patients were excluded, which substantially limited the generalizability of their prognostic model and contributed to inconsistencies with our findings. Similarly, another study identified that patients with persistent lymphopenia had the highest mortality^11^. Yet, this trajectory model was not externally validated, raising concerns about its stability across populations. Additionally, immunosuppressed patients were excluded. In addition, their analysis categorized septic patients into only three classes, whereas our study identified four distinct trajectory classes, providing a more nuanced representation of host immune phenotypes.

In contrast to both prior studies, we included patients with pre-existing immunocompromised—such as individuals with cancer, organ transplants, or HIV—who are at particularly high risk for sepsis and death^22–24^. Their inclusion may explain why the PL phenotype appeared to have the worst prognosis in our study. Moreover, this inclusion enhanced the real-world applicability of our model, as these patients represent a significant portion of the sepsis population.

This study aimed not only to identify high-risk subphenotype through simple lymphocyte counts but also to build a reliable machine learning model for the early prediction of patients with prolonged immune suppression and poor outcomes. The model was then converted into a user-friendly online calculator designed for clinical use. Beyond lymphocyte count, our research identified several key factors crucial for predicting persistent lymphopenia in sepsis patients, including WBC, age, PCT, PLT, and preexisting immunocompromised status. Age is an inherent risk factor for sepsis severity and has been confirmed by many previous studies^25^. The aging process impairs immune function—specifically, reducing T-cell, B-cel^26^, and innate immune cell activity^27^—collectively lowering antigen presentation, phagocytic capacity, and cellular chemotaxis. As a result, elderly patients are more vulnerable to progressing into an immunosuppressed state after sepsis. Besides, preexisting immunocompromised status, which can be seen in patients with prior conditions like organ transplants, chronic steroid use, or cancers, has consistently been linked to a higher risk of developing sepsis-related immune paralysis. Studies indicate that these patients begin with a compromised immune response, which further deteriorates during sepsis, thereby predisposing them to persistent immune deficiency, higher mortality, and recurrent infections^28^. Furthermore, the decrease in WBC in PL patients indicates a state of immunosuppression. This decline results from reduced immune response and less proliferation of immune cells, leading to fewer circulating white blood cells. This decreased cellularity hampers the host’s ability to fight infections effectively. While our model mainly depends on features selected through the Boruta algorithm, all these indicators are easy to interpret clinically. This interpretability demonstrates the value of our online calculator as a practical tool for bedside decision-making.

Sepsis-induced immunosuppression has increasingly been recognized as a key factor influencing patient outcomes^29^. Early clinical monitoring emphasizes inflammatory or infection-related biomarkers such as WBC, CRP, and PCT^28^. However, immune responses in sepsis are highly heterogeneous. Some develop overwhelming hyperinflammation, while others enter a state of predominant immunosuppression, which is often associated with worse long-term outcomes. Early identification of this immunosuppression group remains a major clinical challenge^27^. Recent studies have emphasized the value of circulating immune cell parameters, including LC^28^, neutrophil-to-lymphocyte ratio (NLR)^29^, and monocytic HLA-DR expression (mHLA-DR)^30^, in assessing the immune status of septic patients. Our results support the importance of monitoring LC dynamically. In the PL group, lymphocyte counts stayed persistently low, indicating severe immunosuppression^31^. Consequently, traditional inflammatory markers (WBC, CRP, PCT) did not show significant abnormalities in PL patients. Similarly, patients in the rapidly decreasing lymphocyte (RDL) group also had relatively normal baseline inflammatory markers, yet their declining LC trajectory was strongly linked to poor prognosis. These findings demonstrate that relying solely on baseline inflammatory markers is inadequate to fully understand immune dysfunction in sepsis.

PL patients show coagulation abnormalities, such as lower platelet counts and longer clotting times. In immunosuppression, impaired pathogen clearance keeps the coagulation system active, raising the risk of disseminated intravascular coagulation (DIC). Evidence indicates that DIC occurs more frequently and is more severe in immunosuppressed patients^32^. Collectively, the PL phenotype signals ongoing immunosuppression and a profound immunity-coagulation imbalance, contributing to the high mortality of sepsis.

Although previous studies have reported an association between persistent lymphopenia and mortality in sepsis, our study provides several additional contributions. First, we identified the persistent lymphopenia subgroup at an early stage by integrating lymphocyte counts with routinely available clinical variables reflecting immune, inflammatory, and coagulation status, and translated this approach into a user-friendly web-based calculator to facilitate clinical use. Second, unlike many prior studies, we included patients with preexisting immunocompromised conditions and adjusted for their immunocompromised status, thereby enhancing the real-world applicability of our findings. Finally, the model was derived from a prospective, multicenter cohort and validated in an independent, external cohort, thereby strengthening its generalizability and robustness.

The study had several limitations. First, the generalizability of our findings may be limited by the single-center validation cohort and the exclusively Chinese population of our study. Previous studies have used the MIMIC database for external validation, so we did not repeat that step. Second, our classification relied exclusively on lymphocyte count dynamics and did not incorporate functional tests of lymphocyte subsets or other immune markers. Lymphocyte count was chosen as it is a standard, readily available component of routine blood tests, simplifying the clinical application of our findings. Third, the online prediction tool we developed has not yet been tested prospectively in actual clinical workflows, and future clinical studies are needed to confirm its practical usefulness and clinical value. Fourth, although adding the PL subphenotype produced a small but statistically significant increase in AUC in the external validation cohort, the model’s overall discrimination remained modest, likely due to the substantial biological and clinical heterogeneity of sepsis. Our ICU mortality model was not intended as a definitive bedside tool, but rather to demonstrate that the PL trajectory is a significant prognostic marker and that early identification of this pattern may be valuable. These findings suggest that future ICU mortality prediction models should consider including the PL trajectory as a candidate predictor.

## Conclusion

By applying latent class trajectory modeling (LCTM) to a national, multicenter prospective cohort and an external validation cohort, we identified distinct LC trajectories, revealing the heterogeneity of immune responses in sepsis. The early identification of patients with persistent lymphopenia is crucial for assessing disease severity and prognosis. Future research should focus on developing individualized immunomodulatory strategies tailored to these specific immune subphenotypes.

## Supplementary Information

Supplementary Information.

## Data Availability

The data can be reasonably applied to the corresponding author.

## Abbreviations

LC: Lymphocyte count
PL: Persistent lymphopenia
ICU: Intensive care unit
LCTM: Latent class trajectory modelling
SHAP: SHapley Additive exPlanations
CMS: China Multicenter Sepsis database
PUMCH: Peking Union Medical College Hospital
AIC: Akaike information criterion
BIC: Bayesian information criterion
SIL: Slowly increasing lymphocytes
NL: Normal lymphocytes
RDL: Rapidly declining lymphocytes
HR: Heart rate
RR: Respiratory rate
MAP: Mean arterial pressure
T: Temperature
WBC: White blood cell count
PCT: Procalcitonin
CRP: C-reactive protein
PLT: Platelet count
PT: Prothrombin time
INR: International normalized ratio
APTT: Activated partial thromboplastin time
Fg: Fibrinogen
DD: D-Dimer
FDP: Fibrin degradation products
PaO2/FiO2: Oxygenation index
Lac: Lactic acid
Cr: Creatinine
TBIL: Total bilirubin
OR: Odds ratio
DIC: Disseminated intravascular coagulation
