# Supplementary material for: A machine learning-based prediction model for poor prognosis in sepsis using lymphocyte count: a national, multicenter prospective cohort
Source: Sci Rep. 2026 Jan 22;16:3816. doi: 10.1038/s41598-025-33980-x (PMC12852116; doi:10.1038/s41598-025-33980-x)
Supplement: Supplementary file 1 — Supplementary Information. [file 41598_2025_33980_MOESM1_ESM.docx]

**Supplementary Table 1 Participating centers from the China Multicenter Sepsis (CMS) Database**

| **Participating centers** | **Location** |
| --- | --- |
| The First Hospital of Jilin University | Northeast China |
| The First Hospital of China Medical University | Northeast China |
| The People's Hospital of Liaoning Province | Northeast China |
| The First Affiliated Hospital of Chongqing Medical University | Southwest China |
| The Second Affiliated Hospital of Kunming Medical University | Southwest China |
| Beijing Friendship Hospital, Capital Medical University | North China |
| Tianjin First Central Hospital | North China |
| West China Hospital, Sichuan University | Southwest China |
| The Second Affiliated Hospital, Zhejiang University School of Medicine | East China |
| The First Affiliated Hospital, Zhejiang University School of Medicine | East China |
| The First Affiliated Hospital of Harbin Medical University | Northeast China |
| The First Hospital of Qinhuangdao | North China |
| Union Hospital, Tongji Medical College, Huazhong University of Science and Technology | Central China |
| Qilu Hospital of Shandong University (Qingdao) | East China |
| Beijing Tsinghua Changgung Hospital | North China |
| Henan Provincial People's Hospital | Central China |
| Zhongshan Hospital | South China |
| Shenyang the Fourth Hospital of People | Northeast China |
| The First Affiliated Hospital of Dalian Medical University | Northeast China |
| The Affiliated Hospital of Qingdao University | East China |
| Xiangya Third Hospital | Central China |
| Zhongda Hospital, Southeast University | East China |
| Peking University People's Hospital | North China |
| General Hospital of Ningxia Medical University | Northwest China |
| The Second Affiliated Hospital of Dalian Medical University | Northeast China |
| The First Affiliated Hospital of Kunming Medical University | Southwest China |
| Shengjing Hospital of China Medical University | Northeast China |
|  |  |

**Supplementary Table 2 Missing data in derivation and external validation cohorts**

| Variable n (%) | Derivation Cohort (n=2085) | Validation Cohort (n=1299) |
| --- | --- | --- |
| Sex | 0 | 0 |
| Age | 2 (0.10) | 0 |
| Hypertension | 0 | 0 |
| Diabetes | 0 | 0 |
| Coronary heart disease | 0 | 0 |
| Heart failure | 0 | 0 |
| Respiratory system | 0 | 0 |
| Renal insufficiency | 0 | 0 |
| Liver insufficiency | 0 | 0 |
| Cancer | 0 | 0 |
| Immunocompromised | 0 | 0 |
| Lung | 0 | 0 |
| Abdomen | 0 | 0 |
| Urinary | 0 | 0 |
| Bloodstream | 0 | 0 |
| Skin | 0 | 0 |
| Nervous system | 0 | 0 |
| Others | 0 | 0 |
| Heart rate | 4 (0.19) | 0 |
| Repatriation rate | 8 (0.38) | 342 (26.33) |
| Mean arterial pressure | 56 (2.69) | 8 (0.62) |
| Temperature | 6 (0.29) | 1 (0.08) |
| White blood cell count | 7 (0.34) | 0 |
| Lymphocyte count | 13 (0.62) | 5 (0.38) |
| Procalcitonin | 178 (8.54) | 112 (8.62) |
| C-reactive protein | 271 (13.00) | 375 (28.87) |
| Platelet count | 19 (0.91) | 0 |
| Prothrombin time | 101 (4.84) | 16 (1.23) |
| International normalized ratio | 66 (3.17) | 16 (1.23) |
| Activated partial thromboplastin time | 40 (1.92) | 16 (1.23) |
| Fibrinogen | 52 (2.49) | 16 (1.23) |
| D-dimer | 118 (5.66) | 23 (1.77) |
| Fibrin degradation products | 579 (27.77) | Not Included |
| Oxygenation index | 143 (6.86) | 231 (17.78) |
| Lactic acid | 62 (2.97) | 214 (16.47) |
| Creatinine | 39 (1.87) | 0 |
| Total bilirubin | 59 (2.83) | 0 |
| SOFA | 17 (0.82) | 0 |
| APACHE II | 0 | 235 (18.09) |
| ICU mortality | 0 | 0 |
| Hospital mortality | 0 | 0 |
| 28-day mortality | 0 | 0 |

The number before the parentheses represents the missing cases, and the number inside the parentheses represents the missing rate (in percentage).

**Supplementary Table 3 The clinical characteristics between the derivation cohort and the external validation cohort**

|  | **Derivation cohort (n = 2085)** | **External validation cohort (n = 1299)** | ***P* overall** |
| --- | --- | --- | --- |
| **Demographic characteristics** |  |  |  |
| Sex (male), n (%) | 1351 (64.8) | 810 (62.5) | 0.189 |
| Age (year), median (IQR) | 67.0 (56.0, 74.0) | 62.0 (48.0, 72.0) | <0.001 |
| **Preexisting conditions, n (%)** |  |  |  |
| Hypertension | 842 (40.4) | 588 (45.3) | 0.006 |
| Diabetes | 552 (26.5) | 382 (29.4) | 0.069 |
| Coronary heart disease | 302 (14.5) | 268 (20.6) | <0.001 |
| Chronic obstructive pulmonary disease | 65 (3.1) | 39 (3.0) | 0.931 |
| Cancer | 238 (11.4) | 288 (22.2) | <0.001 |
| Immunocompromised | 293 (14.1) | 161 (12.4) | 0.185 |
| **Infection of site, n (%)** |  |  |  |
| Lung | 894 (42.9) | 693 (53.3) | <0.001 |
| Abdomen | 867 (41.6) | 313 (24.1) | <0.001 |
| Urinary | 126 (6.0) | 54 (4.2) | 0.022 |
| Bloodstream | 16 (0.8) | 44 (3.4) | <0.001 |
| Skin | 82 (3.9) | 27 (2.1) | 0.004 |
| Nervous system | 22 (1.1) | 32 (2.5) | 0.002 |
| Others | 80 (3.7) | 136 (10.5) | <0.001 |
| **Vital signs, median (IQR)** |  |  |  |
| Heart rate (per minute) | 103.0 (88.0, 120.0) | 111.0 (98.0, 126.0) | <0.001 |
| Repatriation rate (per minute) | 20.0 (15.0, 25.0) | 23.0 (18.0, 27.0) | <0.001 |
| Mean arterial pressure (mmHg) | 76.0 (67.0, 89.0) | 68.0 (62.0, 74.0) | <0.001 |
| Temperature (°C) | 37.0 (36.5, 38.0) | 37.7 (37.0, 38.3) | <0.001 |
| **Laboratory parameters, median (IQR)** |  |  |  |
| White blood cell count (10^9^/L) | 11.0 (7.1, 16.4) | 13.0 (9.0, 17.8) | <0.001 |
| Lymphocyte count (10^9^/L) | 0.8 (0.4, 1.1) | 0.6 (0.4, 0.9) | <0.001 |
| Procalcitonin (ng/ml) | 3.8 (0.6, 21.9) | 1.7 (0.4, 8.6) | <0.001 |
| C-reactive protein (mg/L) | 119.0 (55.4, 203.0) | 104.0 (47.3, 188.0) | 0.002 |
| Platelet count (10^9^/L) | 161.0 (96.0, 235.0) | 145.0 (88.0, 199.0) | <0.001 |
| Prothrombin time (Sec) | 15.0 (13.5, 16.9) | 14.4 (13.2, 16.3) | <0.001 |
| International ratio | 1.2 (1.1, 1.4) | 1.2 (1.1, 1.4) | 0.696 |
| Activated partial thromboplastin time (Sec) | 35.2 (29.6, 42.7) | 32.2 (28.3, 38.5) | <0.001 |
| Fibrinogen (g/L) | 4.3 (3.0, 6.0) | 3.9 (3.0, 5.0) | <0.001 |
| D-dimer (μg/ml) | 4.0 (2.1, 8.6) | 4.4 (2.0, 10.3) | 0.045 |
| Oxygenation index (mmHg) | 225.0 (153.0, 311.0) | 217.0 (128.0, 298.0) | <0.001 |
| Lactic acid (mmol/L) | 1.9 (1.3, 3.1) | 2.5 (1.6, 4.1) | <0.001 |
| Creatinine (mg/dL) | 104.0 (64.6, 181.0) | 87.0 (61.0, 159.0) | <0.001 |
| Total bilirubin (μmol/L) | 17.4 (10.9, 29.0) | 16.3 (10.4, 26.9) | 0.018 |
| **Clinical scores, median (IQR)** |  |  |  |
| SOFA | 7.0 (5.0, 10.0) | 8.0 (5.0, 11.0) | <0.001 |
| APACHE II | 14.0 (11.0, 18.0) | 19.0 (14.0, 24.0) | <0.001 |
| **Clinical outcomes, n (%)** |  |  |  |
| ICU mortality | 460 (22.1) | 233 (17.9) | 0.004 |
| Hospital mortality | 512 (24.6) | 263 (20.2) | 0.004 |
| 28-day mortality | 483 (23.2) | 200 (15.4) | <0.001 |

Presented are the comparisons of demographics, preexisting conditions, vital signs, laboratory parameters, clinical scores, and outcomes between the derivation and external validation cohorts. Age, laboratory parameters, vital signs, and clinical scores are presented as median (IQR), and all other values as count (percentage).

**Supplementary Table 4 Fit statistics of LCTM from 1 to 6** **subphenotypes**

| **Number of trajectories** | **Loglik** | | **AIC** | | **BIC** | | **Entropy** | | **AvePP of each trajectory** | **Proportion of each trajectory** |
| --- | --- | --- | --- | --- | --- | --- | --- | --- | --- | --- |
| 1 | -5864.71 | 11743.42 | | 11782.92 | | 1.00000 | | 1.0000 | | 1.0000 |
| 2 | -5602.24 | 11228.47 | | 11296.18 | | 0.5105 | | 0.8271/ 0.8918 | | 0.5631/ 0.4369 |
| 3 | -5276.64 | 10587.27 | | 10683.20 | | 0.7110 | | 0.8936/ 0.8577 / 0.8409 | | 0.6259/ 0.2422/ 0.1319 |
| 4 | -5216.25 | 10476.50 | | 10600.64 | | 0.6392 | | 0.7886 /0.7511/ 0.8691 / 0.8371 | | 0.2825/ 0.4024/ 0.1784/ 0.1367 |
| 5 | -5184.55 | 10423.09 | | 10575.44 | | 0.6289 | | 0.7798 / 0.6976 / 0.8305 / 0.8676 /0.7116 | | 0.1314/ 0.3271/ 0.1501/ 0.1391/ 0.2523 |
| 6 | -5166.13 | 10396.27 | | 10576.83 | | 0.6271 | | 0.7684/0.6795/0.6998/0.6568/0.8475/0.8687 | | 0.1189/0.3338/0.288/0.2398/0.1281/0.1506 |

| **Variables** | **Coefficient** | **Standard error** |
| --- | --- | --- |
| intercept Subphenotype 1 | 0.41033 | 0.02025 |
| intercept Subphenotype 2 | 0.66888 | 0.02053 |
| intercept Subphenotype 3 | 1.00388 | 0.03376 |
| intercept Subphenotype 4 | 1.50716 | 0.05112 |
| Day Subphenotype 1 | -0.01174 | 0.00904 |
| Day Subphenotype 2 | 0.03985 | 0.01268 |
| Day Subphenotype 3 | 0.26231 | 0.01753 |
| Day Subphenotype 4 | -0.38559 | 0.02576 |
| (Day)^2 Subphenotype 1 | 0.00354 | 0.00127 |
| (Day)^2 Subphenotype 2 | -0.00055 | 0.00171 |
| (Day)^2 Subphenotype 3 | -0.02860 | 0.00237 |
| (Day)^2 Subphenotype 4 | 0.05086 | 0.00281 |

**Supplementary Table 5 Fixed effects across four subphenotypes**

|  | **Total (n = 2085)** | **PL (n = 589）** | **SIL(n = 839)** | **NL (n = 372)** | **RDL (n = 285)** | ***P* overall** |
| --- | --- | --- | --- | --- | --- | --- |
| **Vital signs, median (IQR)** |  |  |  |  |  |  |
| Heart rate (per minute) | 103.0 (88.0, 120.0) | 102.0 (89.0, 118.0) | 102.0 (86.2, 118.0) | 102.0 (87.0, 120.0) | 110.0 (90.0, 128.0) | 0.001 |
| Repatriation rate (per minute) | 20.0 (15.0, 25.0) | 20.0 (15.0, 25.0) | 20.0 (15.0, 25.0) | 20.0 (15.0, 25.0) | 20.0 (15.0, 25.0) | 0.783 |
| Mean arterial pressure (mmHg) | 76.0 (67.0, 89.0) | 77.0 (68.0, 89.0) | 76.0 (67.0, 88.0) | 76.0 (67.0, 88.8) | 75.0 (67.0, 89.0) | 0.699 |
| Temperature (°C) | 37.0 (36.5, 38.0) | 37.0 (36.5, 37.9) | 37.0 (36.5, 38.0) | 37.1 (36.5, 37.9) | 37.0 (36.5, 38.0) | 0.661 |
| **Laboratory parameters, median (IQR)** |  |  |  |  |  |  |
| White blood cell count (10^9^/L) | 11.0 (7.1, 16.4) | 8.5 (4.7, 13.0) | 11.1 (7.5, 16.2) | 13.1 (9.4, 19.0) | 14.0 (9.1, 20.1) | <0.001 |
| Lymphocyte count (10^9^/L) | 0.7 (0.4, 1.1) | 0.4 (0.2, 0.5) | 0.7 (0.5, 0.9) | 1.0 (0.7, 1.4) | 1.8 (1.4, 2.5) | <0.001 |
| Procalcitonin (ng/ml) | 3.8 (0.6, 21.9) | 4.0 (0.7, 21.0) | 4.5 (0.9, 23.6) | 2.7 (0.4, 25.8) | 2.3 (0.4, 10.6) | 0.001 |
| C-reactive protein (mg/L) | 119.0 (55.4, 203.0) | 117.0 (55.2, 199.0) | 127.0 (67.4, 211.0) | 115.0 (45.9, 202.0) | 101.0 (43.2, 187.0) | 0.002 |
| Platelet count (10^9^/L) | 161.0 (96.0, 235.0) | 132.0 (78.0, 199.0) | 165.0 (104.0, 234.0) | 172.0 (100.0, 258.0) | 197.0 (121, 292.0) | <0.001 |
| Prothrombin time (Sec) | 15.0 (13.5, 16.9) | 15.0 (13.8, 17.0) | 15.0 (13.8, 17.0) | 14.5 (13.0, 16.3) | 15.0 (13.2, 16.5) | 0.002 |
| International ratio | 1.2 (1.1, 1.4) | 1.3 (1.1, 1.5) | 1.3 (1.1, 1.5) | 1.2 (1.1, 1.4) | 1.2 (1.1, 1.4) | 0.002 |
| Activated partial thromboplastin time (Sec) | 35.2 (29.6, 42.7) | 36.5 (30.6, 43.9) | 35.8 (30.0, 43.2) | 33.9 (28.8, 40.7) | 32.7 (28.1, 40.2) | <0.001 |
| Fibrinogen (g/L) | 4.3 (3.0, 6.0) | 4.1 (2.8, 5.8) | 4.6 (3.1, 6.1) | 4.4 (3.0, 6.1) | 4.0 (2.7, 5.5) | 0.001 |
| D-dimer (μg/ml) | 4.0 (2.1, 8.6) | 3.8 (2.1, 8.4) | 4.2 (2.3, 8.8) | 3.9 (2.0, 9.3) | 3.5 (1.9, 7.6) | 0.017 |
| Fibrin degradation products (μg/mL) | 13.4 (7.0, 26.9) | 11.9 (6.6, 25.0) | 15.3 (8.3, 29.0) | 13.3 (6.1, 28.8) | 11.6 (6.0, 21.5) | 0.002 |
| Oxygenation index (mmHg) | 225.0 (153.0, 311.0) | 204.0 (141.0, 304.0) | 230.0 (161.0, 314.0) | 232.0 (171.0, 313.0) | 230.0 (150.0, 305.0) | 0.021 |
| Lactic acid (mmol/L) | 1.9 (1.3, 3.1) | 1.9 (1.3, 3.1) | 1.9 (1.2, 3.0) | 1.9 (1.3, 3.0) | 1.9 (1.3, 3.2) | 0.585 |
| Creatinine (mg/dL) | 104.0 (64.6, 181.0) | 105.0 (67.0, 176.0) | 108.0 (66.6, 181.0) | 90.5 (57.4, 175.0) | 109.0 (66.0, 189.0) | 0.051 |
| Total bilirubin (μmol/L) | 17.4 (10.9, 29.0) | 17.6 (10.8, 29.5) | 17.9 (11.5, 30.3) | 15.8 (9.9, 28.7) | 16.9 (10.8, 26.0) | 0.117 |

**Supplementary Table 6 Laboratory values among subphenotypes in the derivation cohort**

Presented are the comparisons of vital signs, laboratory parameters in the derivation cohort between subphenotypes: PL (Persistent Lymphopenia), SIL (Slowly Increasing Lymphocyte), NL (Normal Lymphocyte), and RDL (Rapidly Declining Lymphocyte). Vital signs and laboratory parameters are presented as median (IQR).

|  | **Total (n = 1299)** | **PL (n = 343)** | **SIL (n = 527)** | **NL (n = 337)** | **RDL (n = 92)** | ***P* overall** |
| --- | --- | --- | --- | --- | --- | --- |
| **Vital signs, median (IQR)** |  |  |  |  |  |  |
| Heart Rate (per minute) | 111.0 (98.0, 126.0) | 113.0 (99.0, 130.0) | 108.0 (98.0, 123.0) | 110.0 (98.0, 126.0) | 116.0 (103.0, 135.0) | 0.002 |
| Repatriation Rate (per minute) | 23.0 (18.0, 27.0) | 24.0 (19.0, 28.0) | 22.0 (18.0, 27.0) | 23.0 (18.0, 26.0) | 25.0 (20.0, 28.0) | 0.126 |
| Mean Arterial Pressure (mmHg) | 68.0 (62.0, 74.0) | 68.0 (61.0, 74.0) | 69.0 (63.0, 74.5) | 69.0 (63.0, 75.0) | 67.0 (61.8, 74.0) | 0.07 |
| Temperature (°C) | 37.7 (37.0, 38.3) | 37.5 (37.0, 38.2) | 37.7 (37.1, 38.3) | 37.7 (37.1, 38.3) | 38.0 (37.2, 38.6) | 0.012 |
| **Laboratory parameters, median (IQR)** |  |  |  |  |  |  |
| White blood cell count (10^9^/L) | 13.0 (9.0, 17.8) | 10.2 (6.2, 16.4) | 12.7 (9.5, 17.8) | 14.6 (11.1, 19.5) | 15.5 (11.5, 19.5) | <0.001 |
| Lymphocyte count (10^9^/L) | 0.6 (0.4, 0.9) | 0.3 (0.2, 0.5) | 0.6 (0.4, 0.8) | 0.8 (0.6, 1.2) | 1.3 (1.0, 1.6) | <0.001 |
| Procalcitonin (ng/ml) | 1.7 (0.4, 8.6) | 1.7 (0.5, 11.0) | 1.7 (0.3, 7.2) | 1.8 (0.3, 9.2) | 1.8 (0.3, 6.4) | 0.404 |
| C-reactive protein (mg/L) | 104.0 (47.3, 188.0) | 92.6 (46.8, 190.0) | 115.0 (51.8, 196.0) | 101.0 (44.4, 183.0) | 81.1 (43.4, 154.0) | 0.160 |
| Platelet count (10^9^/L ) | 145.0 (88.0, 199.0) | 104.0 (48.5, 160.0) | 151.0 (100.0, 200.0) | 169.0 (113.0, 232.0) | 152.0 (87.5, 203.0) | <0.001 |
| Prothrombin time (Sec) | 14.4 (13.2, 16.3) | 14.9 (13.3, 17.2) | 14.3 (13.2, 15.8) | 14.2 (13.2, 15.9) | 14.6 (13.3, 16.8) | 0.005 |
| International ratio | 1.2 (1.1, 1.4) | 1.3 (1.1, 1.5) | 1.2 (1.1, 1.4) | 1.2 (1.1, 1.4) | 1.3 (1.1, 1.5) | 0.014 |
| Activated partial thromboplastin time (Sec) | 32.2 (28.3, 38.5) | 33.9 (29.9, 41.2) | 31.6 (27.8, 37.8) | 31.3 (27.7, 36.3) | 32.4 (29.2, 41.2) | <0.001 |
| Fibrinogen (g/L) | 3.9 (3.0, 5.0) | 3.7 (2.6, 4.7) | 4.0 (3.0, 5.0) | 4.2 (3.1, 5.2) | 3.8 (2.8, 4.9) | 0.001 |
| D-dimer (μg/ml) | 4.4 (2.0, 10.3) | 5.7 (2.8, 13.5) | 4.3 (2.0, 10.2) | 3.4 (1.4, 7.1) | 6.0 (2.0, 11.8) | <0.001 |
| Oxygenation index (mmHg) | 217.0 (128.0, 298.0) | 172.0 (95.4, 260.0) | 230.0 (134.0, 309.0) | 241.0 (167.0, 312.0) | 194.0 (139.0, 278.0) | <0.001 |
| Lactic acid (mmol/L) | 2.5 (1.6, 4.1) | 2.5 (1.6, 4.2) | 2.5 (1.6, 3.9) | 2.4 (1.6, 4.1) | 2.8 (1.7, 4.2) | 0.739 |
| Creatinine (mg/dL) | 87.0 (61.0, 159.0) | 100.0 (59.0, 227.0) | 86.0 (61.0, 142.0) | 82.0 (60.0, 125.0) | 101.0 (67.5, 190.0) | 0.021 |
| Total bilirubin (μmol/L) | 16.3 (10.4, 26.9) | 16.9 (10.5, 30.6) | 16.5 (10.7, 26.6) | 14.8 (9.5, 23.0) | 18.9 (13.0, 30.1) | 0.019 |
|  |  |  |  |  |  |  |

**Supplementary Table 7 Laboratory** **values among subphenotypes in the external validation cohort**

The comparisons of vital signs and laboratory parameters among subphenotypes in the external validation cohort are presented. Vital signs and laboratory parameters are presented as median (IQR).

**Supplementary Table 8 Hyperparameters for six models**

| Model | Hyperparameter |
| --- | --- |
| Logistic Regression (LR) | C: 10.0,' penalty: l2, solver: liblinear |
| Random Forest (RF) | max_depth: 8, max_features: 5, min_samples_split: 20, n_estimators: 300 |
| Support Vector Machine (SVM) | C: 0.14290404770224222, gamma: scale |
| Extreme Gradient Boosting (Xgboost) | colsample_bytree: 0.5, learning_rate: 0.03655677584818631, max_depth: 3, subsample: 0.5 |
| Light Gradient Boosting Machine (LightGBM) | learning_rate: 0.01, n_estimators: 300, num_leaves: 20 |
| Multilayer Perceptron (Mlp) | hidden_layer_sizes: 182, learning_rate_init: 0.001306143370306966 |

**Supplementary Table 9** **Detailed performance metrics of various machine learning models for predicting the PL patients across training, test, and external validation sets.**

| **Predictive models** | **Accuracy** | **Precision** | **Sensitivity** | **Specificity** | **AUC** | **F1-score** |
| --- | --- | --- | --- | --- | --- | --- |
| **Training set** | | | | | | |
| LR | 0.764 | 0.785 | 0.230 | 0.975 | 0.816 | 0.356 |
| RF | 0.931 | 0.926 | 0.822 | 0.974 | 0.983 | 0.871 |
| SVM | 0.729 | 0.840 | 0.051 | 0.996 | 0.794 | 0.096 |
| XGB | 0.863 | 0.810 | 0.674 | 0.937 | 0.932 | 0.736 |
| LightGBM | 0.902 | 0.896 | 0.737 | 0.966 | 0.966 | 0.809 |
| Mlp | 0.808 | 0.646 | 0.708 | 0.847 | 0.880 | 0.675 |
| **Test set** | | | | | | |
| LR | 0.762 | 0.759 | 0.232 | 0.975 | 0.816 | 0.355 |
| RF | 0.791 | 0.655 | 0.548 | 0.974 | 0.841 | 0.597 |
| SVM | 0.728 | 0.818 | 0.051 | 0.996 | 0.770 | 0.096 |
| XGB | 0.783 | 0.645 | 0.514 | 0.938 | 0.839 | 0.572 |
| LightGBM | 0.781 | 0.647 | 0.497 | 0.967 | 0.829 | 0.562 |
| Mlp | 0.773 | 0.594 | 0.627 | 0.847 | 0.832 | 0.610 |
| **External validation set** | | | | | | |
| LR | 0.774 | 0.721 | 0.234 | 0.975 | 0.806 | 0.353 |
| RF | 0.792 | 0.590 | 0.693 | 0.974 | 0.848 | 0.63 |
| SVM | 0.753 | 0.806 | 0.085 | 0.996 | 0.769 | 0.153 |
| XGB | 0.774 | 0.561 | 0.658 | 0.938 | 0.835 | 0.606 |
| LightGBM | 0.788 | 0.589 | 0.649 | 0.967 | 0.840 | 0.618 |
| Mlp | 0.762 | 0.538 | 0.687 | 0.847 | 0.821 | 0.603 |

**
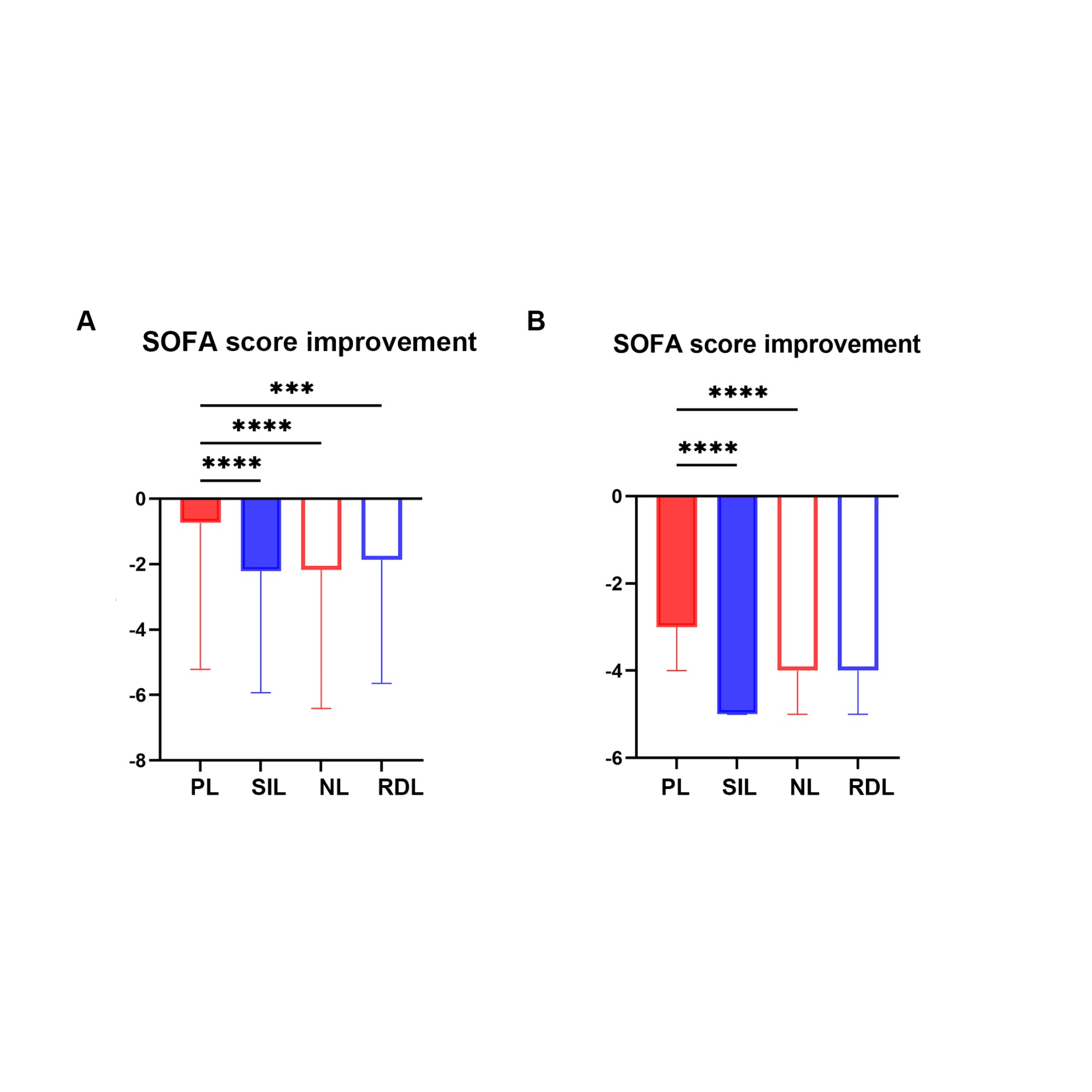
** **Supplementary Figure 1**

**Suppl. Figure 1** The levels of SOFA score improvement represent the mean and standard error (SEM) for each subphenotype. (A) displays the derivation cohort, (B) shows the external validation cohorts.


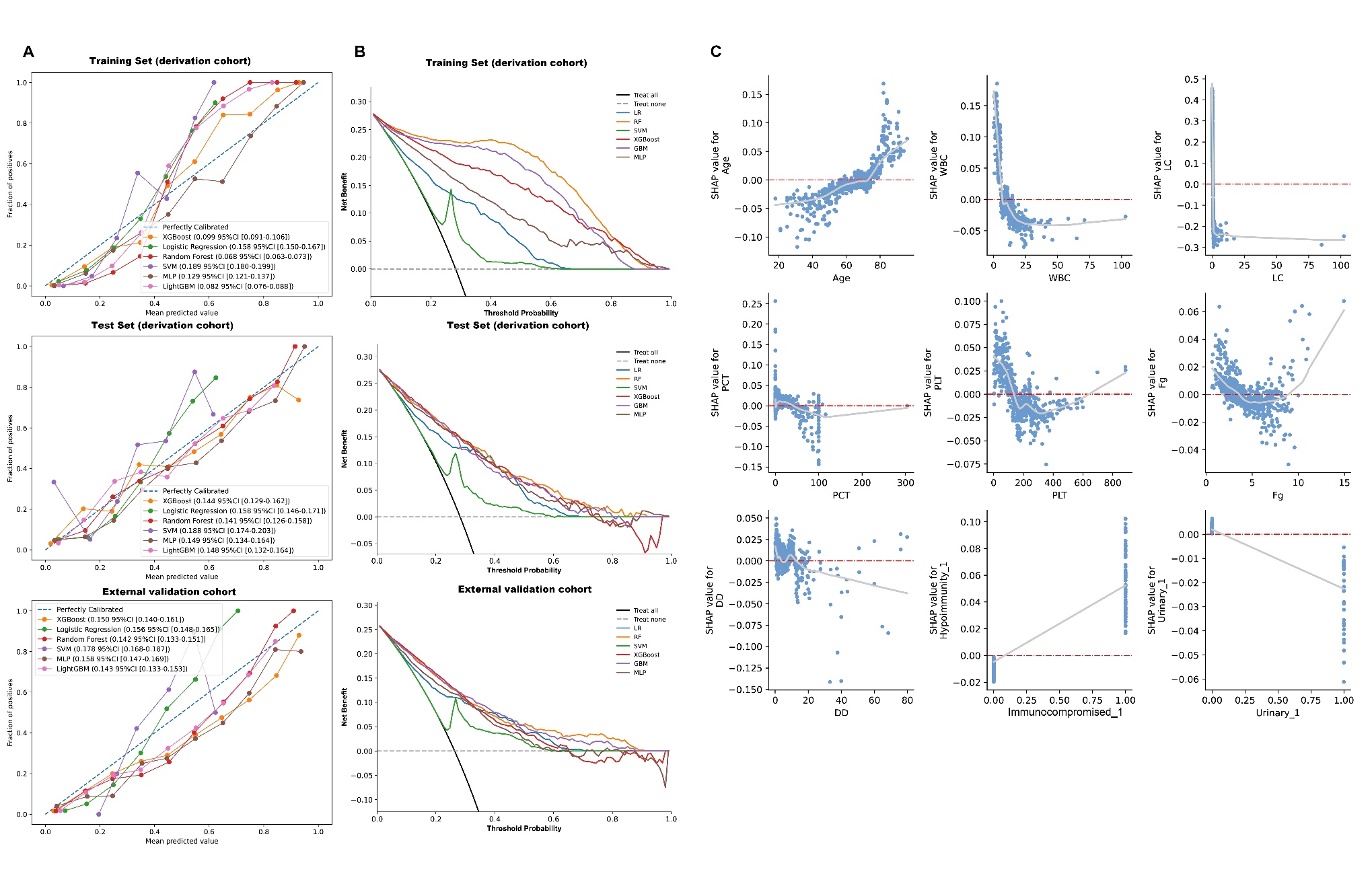
 **Supplementary** **Figure 2**

**Suppl. Figure 2** (A) Calibration Curve for the Training Set, Calibration Curve for the Test Set, Calibration Curve for the External validation Set; (B) Decision Curve Analysis for the Training Set, Decision Curve Analysis for the Test Set, Decision Curve Analysis for the External validation Set. (C) SHAP dependence plot. Each dependence plot shows how a single feature affects the model’s output, with each point representing a patient. SHAP values are on the y-axis, and actual feature values are on the x-axis.


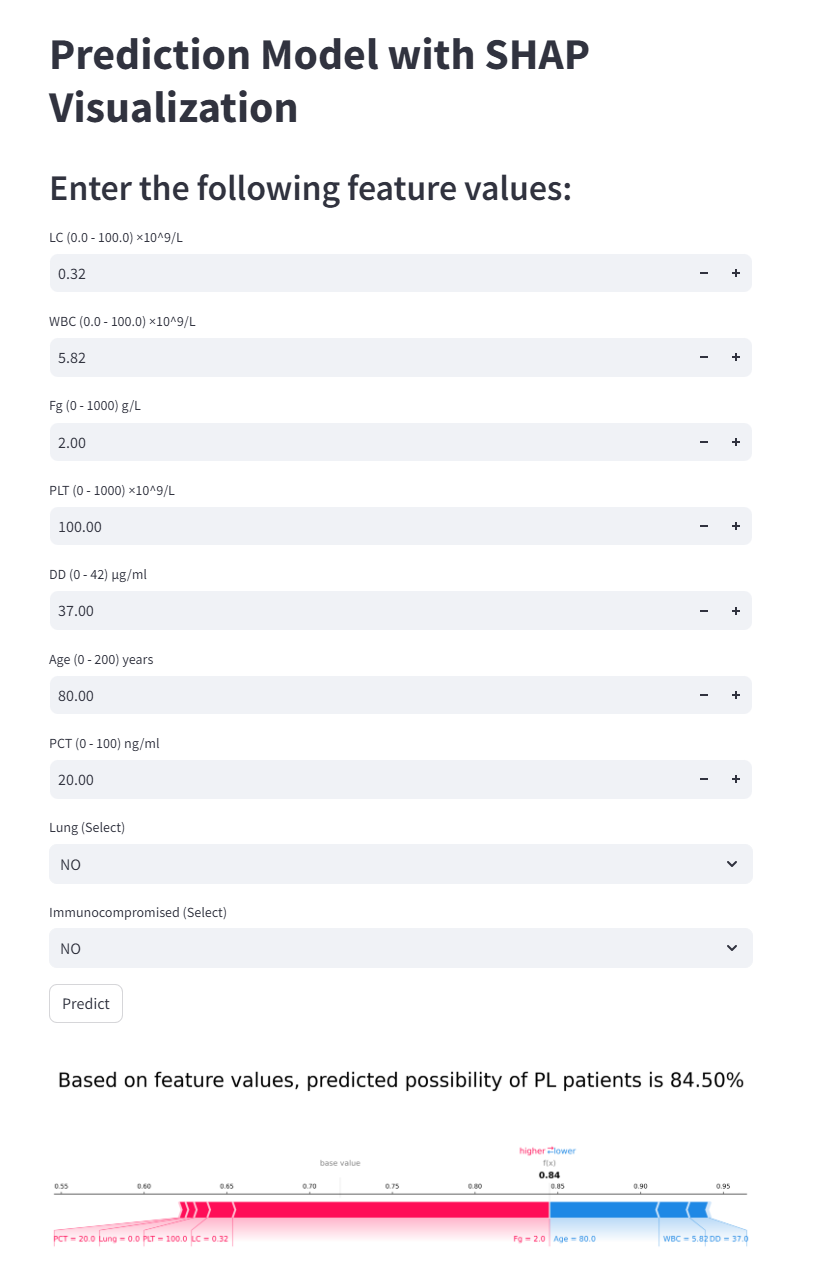
 **Supplementary Figure 3**

**Suppl. Figure 3** An example of a web-based tool. Predicting the PL patients by inputting nine clinical parameters.
